# Effect of acute kidney injury and overall survival in patients with postoperative head and neck cancer who received chemoradiotherapy with cisplatin: A supplementary analysis of the phase II/III trial of JCOG1008

**DOI:** 10.1002/cam4.70235

**Published:** 2024-09-30

**Authors:** Yoshinori Imamura, Naomi Kiyota, Makoto Tahara, Takeshi Kodaira, Ryuichi Hayashi, Hiroshi Nishino, Yukinori Asada, Hiroki Mitani, Shigemichi Iwae, Naoki Nishio, Yusuke Onozawa, Nobuhiro Hanai, Akira Ohkoshi, Hiroki Hara, Nobuya Monden, Masato Nagaoka, Shujiro Minami, Ryo Kitabayashi, Keita Sasaki, Akihiro Homma, the Head and Neck Cancer Study Group of the Japan Clinical Oncology Group (JCOG‐HNCSG)

**Affiliations:** ^1^ Kobe University Hospital Kobe Japan; ^2^ National Cancer Center Hospital East Kashiwa Japan; ^3^ Aichi Cancer Center Hospital Nagoya Japan; ^4^ Jichi Medical University Hospital Shimotsuke Japan; ^5^ Miyagi Cancer Center Natori Japan; ^6^ Cancer Institute Hospital Tokyo Japan; ^7^ Hyogo Cancer Center Akashi Japan; ^8^ Nagoya University Hospital Nagoya Japan; ^9^ Shizuoka Cancer Center Shizuoka Japan; ^10^ Tohoku University Hospital Sendai Japan; ^11^ Saitama Cancer Center Kitaadachi‐gun Japan; ^12^ National Hospital Organization Shikoku Cancer Center Matsuyama Japan; ^13^ Jikei University Hospital Tokyo Japan; ^14^ National Hospital Organization Tokyo Medical Center Tokyo Japan; ^15^ Japan Clinical Oncology Group Data Center/Operations Office National Cancer Center Hospital Tokyo Japan; ^16^ Hokkaido University Hospital Sapporo Japan

**Keywords:** acute kidney injury, cisplatin, head and neck cancer, overall survival

## Abstract

**Background:**

In a randomized phase II/III trial (JCOG1008), weekly cisplatin (40 mg/m^2^) was non‐inferior to 3‐weekly cisplatin (100 mg/m^2^) for postoperative high‐risk head and neck cancer. We investigated how acute kidney injury (AKI), a major dose‐limiting toxicity effect of cisplatin, affects overall survival (OS).

**Methods:**

We analyzed 251 patients from JCOG1008 receiving chemoradiotherapy. AKI was defined based on AKI Network criteria (serum creatinine increase of ≥0.3 mg/dL or ≥1.5‐fold [≥ stage I]) within 30 days after completing chemoradiotherapy. OS in the two arms was compared according to AKI development using the log‐rank test.

**Results:**

The total incidence of AKI was lower in the weekly arm than in the 3‐weekly arm (38/122 [31.1%] vs. 56/129 [43.4%]). Additionally, stage II/III AKI occurred less frequently in the weekly arm than in the 3‐weekly arm (8/122 [6.6%] vs. 19/129 [14.7%]). Cisplatin doses were similar in the weekly arm for patients with and without AKI (median, 238.6 mg/m^2^ vs. 239.2 mg/m^2^; *p* = 0.94), but lower in the 3‐weekly arm for those who developed AKI (median, 276.3 mg/m^2^ vs. 297.4 mg/m^2^; *p* = 0.007). In the weekly arm, there was no difference in OS between patients with and without AKI (hazard ratio [HR], 1.06; 95% confidence interval [CI], 0.53 to 2.10). However, in the 3‐weekly arm, patients with AKI had poorer OS than those without AKI (HR, 1.83; 95% CI, 1.04 to 3.21).

**Conclusions:**

In this supplementary analysis of JCOG1008 data, AKI impacted the OS of patients with head and neck cancer undergoing postoperative chemoradiotherapy in the 3‐weekly arm but not in the weekly arm. Our results further endorse the utilization of weekly cisplatin at 40 mg/m^2^ in this setting.

## Graphical abstract

Weekly administration can improve kidney safety, ensure the maintenance of cisplatin treatment intensity, and consequently improve overall survival of patients with head and neck cancer who underwent postoperative chemoradiotherapy. Our results provide additional support for the weekly use of cisplatin at a dose of 40 mg/m^2^ in this setting.

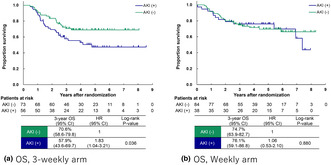

## 1 INTRODUCTION

Head and neck squamous cell carcinoma (HNSCC) accounts for approximately 4% of all cancer cases in the United States, with an estimated 66,470 new diagnoses and 15,050 deaths in 2022.^1^ Effective early screening methods have not yet been established and more than half of cases are diagnosed at an advanced stage.^2^ In cases of individuals with locally advanced HNSCC, cisplatin‐based chemoradiotherapy (CRT) has become the standard treatment, both as a postoperative treatment for patients with a high risk of recurrence and an alternative to surgical intervention.^3^

In terms of combination regimens, high‐dose cisplatin is considered the established standard because of its reliable efficacy.^4^, ^5^ However, a significant issue associated with this approach is the increased incidence of acute kidney injury (AKI), which is a major dose‐limiting side effect of cisplatin.^6^ The cumulative cisplatin dose is of pivotal importance as a prognostic factor for CRT.^7^, ^8^, ^9^, ^10^, ^11^ Consequently, there are concerns about sustaining treatment adherence, which has led to a narrowing of the eligible patient population.^12^ Some reports suggest that the occurrence of AKI is related to the cumulative dose of cisplatin and affects the long‐term prognosis.^13^, ^14^ Thus, efforts have been directed toward the development of regimens with reduced renal toxicity.^15^, ^16^

Recently, a randomized phase II/III trial of JCOG1008 has demonstrated that CRT with weekly cisplatin at 40 mg/m^2^ (weekly arm) (Day 1, 8, 15, 22, 29, 36, 43) is non‐inferior to that with 3‐weekly cisplatin at 100 mg/m^2^ (3‐weekly arm) (Day 1, 22, 43) in terms of overall survival (OS) in patients with postoperative high‐risk HNSCC, with a hazard ratio (HR) of 0.69 (99.1% confidence interval [CI], 0.37 to 1.27 < 1.32) and favorable acute safety profile.^17^ However, the underlying factors contributing to this trend have not been sufficiently investigated, although the frequency and severity of AKI may have influenced the results.

The aim of this post hoc analysis was to evaluate the degree of AKI severity and treatment adherence and their influence on the long‐term prognosis of patients diagnosed with HNSCC who received postoperative CRT. This analysis was stratified based on the method of cisplatin administration, using data from the JCOG1008 dataset.

## 2 METHODS

### 2.1 Participants and exposure

In total, 251 patients from 28 institutions participated in JCOG1008, which was registered with the Japan Registry of Clinical Trials (jRCTs031180135) and approved by the National Cancer Center Hospital–Certified Review Board (CRB3180008). The detailed design and procedural aspects of this phase II/III trial have been published previously.^18^

JCOG1008 was designed to assess the noninferiority of concurrent CRT with weekly cisplatin at 40 mg/m^2^ (weekly arm) to concurrent CRT with 3‐weekly cisplatin at 100 mg/m^2^ (3‐weekly arm) for postoperative high‐risk patients with locally advanced HNSCC. Creatinine clearance (CCr) estimated using the Cockcroft‐Gault equation ≥60 mL/min was set as an eligibility criterion. Cisplatin was administered according to the dose reduction or discontinuation criteria shown in Table S1. Radiotherapy was delivered using high‐energy photons ranging from 4 to 10 MV x‐rays, with a cumulative dose of 66 Gy in 33 fractions spanning a duration of 6.5 weeks.

### 2.2 Outcomes

The primary endpoint in the current supplementary analysis was OS, which was defined as the number of days from randomization to death from any cause and censored on the last day when the patient was alive. Secondary endpoints included relapse‐free survival (RFS) and the proportion of treatment completion. RFS was defined as the number of days from randomization to any disease relapse or death from any cause, and was censored on the last day without an event. Treatment completion was defined as follows: for the 3‐weekly arm, completion of radiotherapy within 66 days and administration of two or three courses of 3‐weekly cisplatin during radiotherapy or within 14 days from the day of completion of radiotherapy; for the weekly arm, completion of radiotherapy within 66 days and administration of five of seven courses of weekly cisplatin during radiotherapy.^18^

AKI was defined as a serum creatinine increase ≥0.3 mg/dL from registration to peak measurements during the treatment period or within 30 days after completion, as per the nephrotoxicity criteria of the National Cancer Institute.^19^ AKI severity was calculated using the AKI Network criteria: stage I, serum creatinine increase ≥0.3 mg/dL or 1.5‐ to 2‐fold above baseline; stage II, serum creatinine increase exceeding 2.0‐ to 3.0‐fold from baseline; stage III, serum creatinine increase exceeding 3.0‐fold from baseline or a serum creatinine level ≥4.0 mg/dL with an acute increase ≥0.5 mg/dL or the requirement for renal replacement therapy.^20^ During the period starting from the initiation of the protocol treatment up to 30 days after completion, adverse events (AEs) data were monitored at least once a week and changes in CCr were calculated using the Cockcroft‐Gault equation.

### 2.3 Statistical analysis

Demographic and baseline patient characteristics were compared between the treatment arms using Fisher's exact test for categorical variables and the Wilcoxon rank‐sum test for continuous variables. OS and RFS based on the presence or absence of AKI were estimated using the Kaplan–Meier method. HRs were calculated using the Cox regression model and the 95% confidence intervals (CIs).

This supplementary analysis was performed for exploratory purposes, and no sample size was predefined. The tests were two‐sided, and statistical significance was set at *p* < 0.05. Statistical analyses were performed using SAS 9.4 (SAS Institute, Cary, NC, USA).

## 3 RESULTS

### 3.1 Patient characteristics

Between October 2012 and December 2018, 261 patients were registered in JCOG1008, with 129 patients receiving 3‐weekly cisplatin plus radiotherapy and 122 receiving weekly cisplatin plus radiotherapy. The treatment arms were generally balanced with respect to the demographic and clinical characteristics after randomization (Table 1).

**TABLE 1:** Patient characteristics.

	3‐weekly arm no (%) *n* = 129	Weekly arm no (%) *n* = 122	*p*‐value^a^
Age (year), median (range)	62 (26–75)	61.5 (20–75)	0.374
Male	107 (83.0)	103 (84.4)	0.865
*ECOG‐performance status*			
0	91 (70.5)	70 (73.0)	0.677
1	38 (29.5)	24 (27.1)	
*Primary site*			
Oral cavity	59 (45.7)	56 (45.9)	0.685
Oropharynx	14 (10.9)	19 (15.6)	
Hypopharynx	44 (34.1)	39 (29.5)	
Larynx	12 (9.3)	11 (9.0)	
*Pathological stage*			
III	9 (7.0)	11 (9.0)	0.628
IVA	114 (88.4)	107 (87.7)	
IVB	5 (3.9)	2 (1.6)	
Unknown	1 (0.8)	2 (1.6)	
Diabetes mellites	18 (14.0)	14 (11.5)	0.576
Hypertension	35 (27.1)	39 (32.0)	0.410
*Main nutritional method*			
Oral	112 (86.8)	116 (95.1)	0.034
Percutaneous endoscopic gastrostomy tube	14 (10.9)	6 (4.9)	
Nasogastric tube	3 (2.3)	0	
Total parenteral nutrition	0	0	
Other	0	0	
*Base‐line laboratory data, median* (*range*)			
Serum creatinine (mg/dL)	0.72 (0.32–1.01)	0.74 (0.44–1.16)	0.155
Serum albumin (g/dL)	3.9 (2.7–4.8)	3.9 (2.5–4.9)	0.839
Serum potassium (mEg/L)	4.2 (3.4–5.2)	4.2 (3.0–6.0)	0.348
Serum magnesium (mEg/L)	2.1 (1.2–2.6)	2.0 (1.5–2.7)	0.091
Creatinine clearance (mL/min)	84.5 (60.7–214.2)	84.8 (60.0–157.2)	0.913

Abbreviation: ECOG, Eastern Cooperative Oncology Group.

^a^ Two‐sided *p*‐value by Wilcoxon's rank‐sum test or Fisher's exact test.

### 3.2 AKI according to cisplatin administration

**FIGURE 1:**
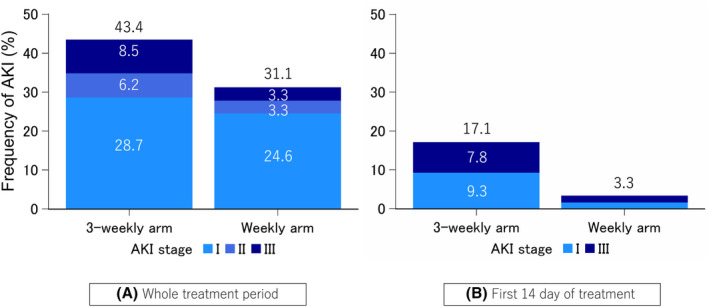
Incidence of acute kidney injury (A) during the entire treatment period and (B) in the first 14 d of treatment.

The total incidence of AKI in the weekly arm was lower than that in the 3‐weekly arm (38/122 [31.1%] vs. 56/129 [43.4%]; *p* = 0.051; Figure 1A). The same trend was observed for the incidence of stage II/III AKI (8/122 [6.6%] vs. 19/129 [14.7%]; *p* = 0.042). In addition, the tendency for a reduced occurrence of AKI in the weekly arm was more pronounced than that in the 3‐weekly arm (4/122 [3.3%] vs. 22/129 [17.1%]; *p* < 0.001) when examining the first 14 days of the treatment period (Figure 1B).

**FIGURE 2:**
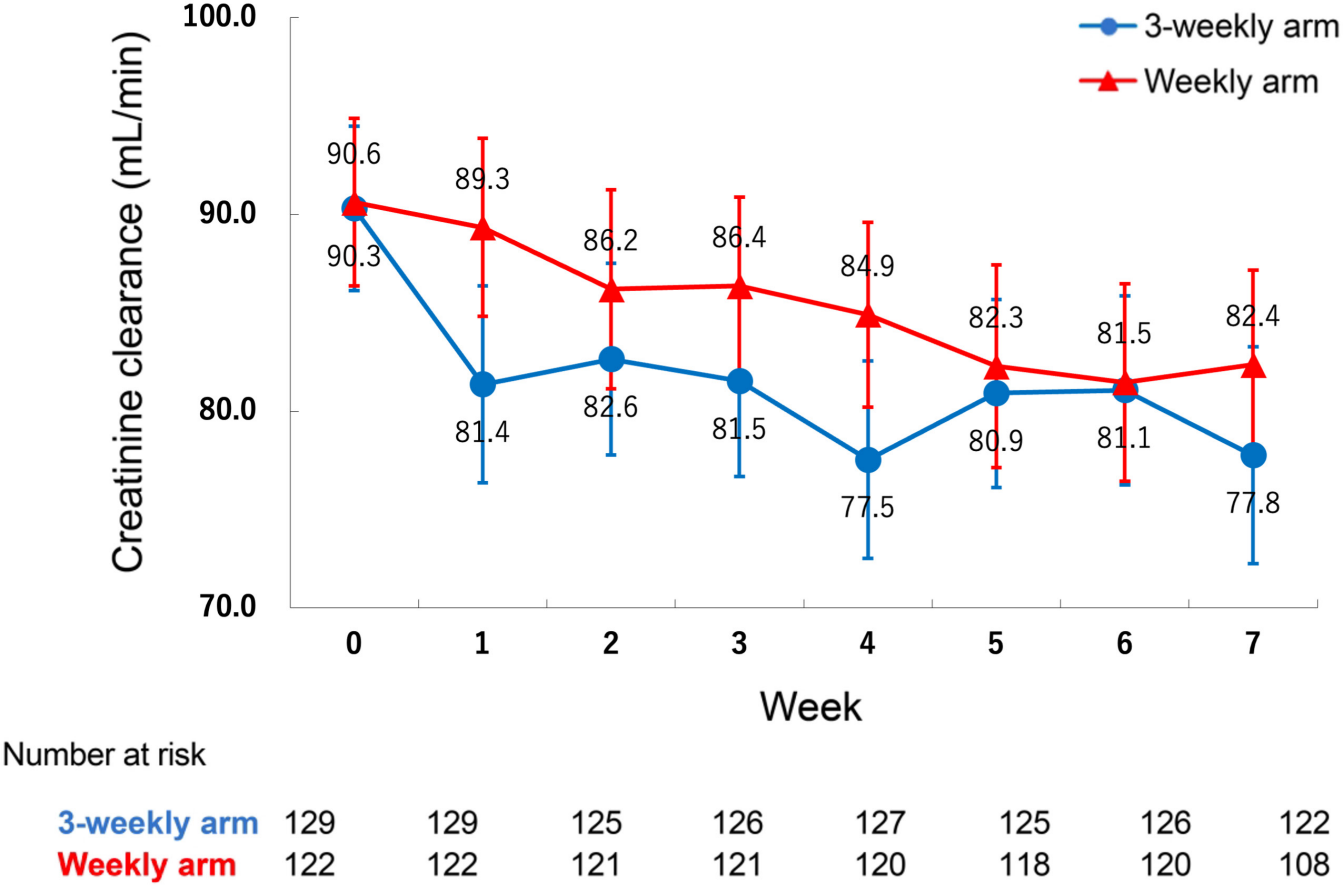
Changes in creatinine clearance (average) during the treatment period. The bars represent the 95% confidence intervals.

The actual weekly changes in the CCr levels are shown in Figure 2. Particularly, in the 3‐weekly arm, CCr declined early in the treatment period and did not recover.

### 3.3 Treatment delivery, compliance, and long‐term outcomes according to cisplatin administration

Treatment delivery and compliance with chemoradiotherapy were adequate and as scheduled in the weekly arm, regardless of the complications of AKI. This included a median total cisplatin dose and proportion of actual to planned delivery of cisplatin of 239 mg/m^2^ and 86%, respectively, in both cases (Table 2). Conversely, in the 3‐weekly arm, these values were significantly lower than in the weekly arm due to the occurrence of AKI, with median values of 297 mg/m^2^ versus 276 mg/m^2^ (*p* = 0.007) and 99% versus 92% (*p* = 0.009) in the cases without and with AKI, respectively. No clinical impact of AKI on the treatment duration and radiotherapy dose was observed in either arm.

**TABLE 2:** Treatment delivery and compliance.

	3‐weekly arm			Weekly arm		
	AKI (−) *n* = 73	AKI (+) *n* = 56	*p*‐value^a^	AKI (−) *n* = 84	AKI (+) *n* = 38	*p*‐value^a^
Total RT dose (Gy), median (IQR)	66 (66–66)	66 (66–66)	0.721	66 (66–66)	66 (66–66)	0.682
Duration of RT (d), median (IQR)	49 (47–50)	50 (47–52)	0.601	49 (46–50)	49 (47–50)	0.369
Interval from surgery to RT initiation (d), median (IQR)	49 (43–55)	48 (39–54.5)	0.187	48 (41.5–55)	50.5 (43–55)	0.153
Cycles of cisplatin, median (IQR)	3 (3–3)	3 (3–3)	0.283	6 (5–7)	7 (6–7)	0.464
Total cisplatin dose (mg/m^2^, IQR)	297.4 (258.9–299.1)	276.3 (219.7–298.0)	0.007	239.2 (199.5–276.7)	238.6 (197.3–277.2)	0.938
Proportion of actual to planned delivery of cisplatin (%), median (IQR)	99.2 (86.4–100.0)	92.4 (73.3–99.9)	0.009	85.7 (71.4–100.0)	85.7 (71.4–100.0)	0.834
Proportion of treatment completion (%), median (95% CI)	94.5 (86.6–98.5)	96.4 (87.7–99.6)	0.697	90.5 (82.1–95.8)	94.7 (82.3–99.4)	0.723

Abbreviations: AKI, acute kidney injury; CI, confidence interval; IQR, interquartile range; RT, radiotherapy.

^a^ Two‐sided *p*‐value by Wilcoxon's rank‐sum test or Fisher's exact test.

**FIGURE 3:**
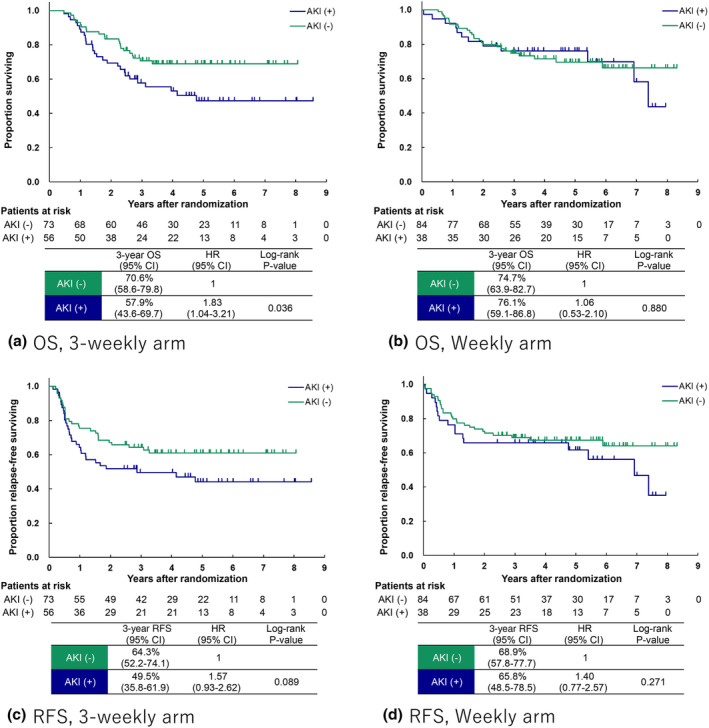
Kaplan–Meier curves according to presence or absence of acute kidney injury. Overall survival in the (A) 3‐weekly arm and (B) weekly arm. Recurrence‐free survival in the (C) 3‐weekly arm and (D) weekly arm.

The median follow‐up period was 3.7 years (4.9 years for survivors). The Kaplan–Meier curves of OS were close to each other by the presence or absence of AKI in the weekly arm (HR, 1.06; 95% CI, 0.53–2.10; *p* = 0.88) but were separated in the 3‐weekly arm (HR, 1.83; 95% CI, 1.04–3.21; *p* = 0.033; Figure 3). Similarly, the Kaplan–Meier curves of RFS were close to each other by the presence or absence of AKI in the weekly arm (HR, 1.40; 95% CI, 0.77–2.57; *p* = 0.27) but were separated in the 3‐weekly arm (HR, 1.57; 95% CI, 0.93–2.62; *p* = 0.089). These trends in the 3‐weekly arm were confirmed regardless of AKI stage.

## 4 DISCUSSION

This supplementary study of JCOG1008 revealed three major findings: (1) less frequent and less severe AKI was observed in the weekly arm than in the 3‐weekly arm among patients diagnosed with HNSCC who underwent cisplatin‐based postoperative CRT; (2) the total cisplatin dose was similar irrespective of the development of AKI in the weekly arm, but was lower in cases of AKI development in the 3‐weekly arm; (3) OS did not differ irrespective of the development of AKI in the weekly arm, but was poorer in the case of AKI in the 3‐weekly arm. In the 3‐weekly arm, the frequency of moderate‐to‐severe AKI was higher, often leading to the need for cisplatin dose reduction or discontinuation. As a consequence, a reduction in the cumulative dose and dose intensity of cisplatin may contribute to a decline in treatment outcomes in cases of AKI. Conversely, in the weekly arm, the risk of moderate‐to‐severe AKI was lower and there were fewer instances of the need to reduce or discontinue cisplatin. Consequently, the cumulative dose and dose intensity of cisplatin were effectively maintained even in cases of AKI, potentially leading to improved treatment outcomes.

Although several trials have assessed the potential of the weekly cisplatin regimen as an alternative to the 3‐weekly regimen, results in the literature are conflicting. The first was a single‐center randomized controlled trial (RCT) conducted in India^21^ that primarily included postoperative cases (93%), although some cases were locally advanced. The aim of that study was to establish the non‐inferiority of weekly cisplatin at a dose of 30 mg/m^2^ to 3‐weekly cisplatin. The primary endpoint was locoregional control, and the study yielded a negative result with an HR of 1.76 (95% CI, 1.11–2.79). The second study was the JCOG1008 trial, a multicenter RCT conducted in Japan.^17^ The aim of that study was to establish the non‐inferiority of weekly cisplatin at a dose of 40 mg/m^2^ to 3‐weekly cisplatin in postoperative high‐risk cases. The primary endpoint was OS, and the study demonstrated non‐inferiority with an HR of 0.69 (99.1% CI, 0.37–1.27 [<1.32]). The third study was the ConCERT trial, a multicenter RCT conducted in India.^22^ The aim of that study was to establish the non‐inferiority of weekly cisplatin at a dose of 40 mg/m^2^ to 3‐weekly cisplatin in non‐surgical locally advanced cases. The primary endpoint, the 2‐year locoregional control rate, demonstrated non‐inferiority with an absolute difference of 3.84% (one‐sided 95% CI = −6.15, 13.80), falling within the pre‐defined non‐inferiority margin of −10%. In the first Indian RCT, whether the efficacy of weekly administration is inferior or a dose of 30 mg/m^2^ is suboptimal remains unclear. The dosage intensity of 40 mg/m^2^/week in the weekly arm is higher than the equivalent dosage intensity of 33 mg/m^2^/week in the 3‐weekly arm, the latter of which is derived from the dosage of 100 mg/m^2^ administered on a once‐every‐3‐weeks schedule. Conversely, the dosage intensity of 30 mg/m^2^/week in the first Indian RCT is lower than 33 mg/m^2^/week. Furthermore, the only negative trial that combined single‐agent cisplatin with radiotherapy in a meta‐analysis of chemotherapy in head and neck cancer utilized a weekly dose of 20 mg/m^2^. These results indicate the significance of dose intensity in cisplatin‐based CRT in patients with HNSCC.

To reduce the toxicities associated with the bolus administration of high‐dose cisplatin, various dosing schedules have been investigated, including weekly, daily, and fractionated dosing over 4–5 days.^15^, ^16^, ^23^, ^24^, ^25^, ^26^ The aim of these modifications is to improve treatment compliance and enhance the radiosensitizing effect.^27^, ^28^ In patients with HNSCC undergoing cisplatin‐based CRT, the total cumulative cisplatin dose is a well‐established factor contributing to improved survival outcomes.^7^, ^8^, ^9^, ^10^, ^11^ Several studies have suggested that an additional effect of CRT on RT can be expected when the cumulative dosage exceeds 200 mg/m^2^, regardless of dosing schedules.^8^, ^9^, ^29^ Nevertheless, this supplementary analysis of the JCOG 1008 trial revealed that in cases wherein AKI occurred in the 3‐weekly arm, the median dosage reached 276 mg/m^2^, with the lower quantile remaining at 220 mg/m^2^. This suggests that a significant proportion of the patients received doses exceeding 200 mg/m^2^. Moreover, the median dose in the 3‐weekly arm, even in cases wherein AKI occurred, was higher than that in the weekly arm (approximately 240 mg/m^2^ regardless of the development of AKI), which is difficult to explain solely based on the cumulative cisplatin dose. In this context, it was noteworthy that the estimated dose intensity in JCOG 1008 was higher in the weekly than in the 3‐weekly cisplatin arm (33.6 mg/m^2^ vs. 29.3 mg/m^2^), although the mean proportion of actual to planned delivery of cisplatin was lower in the weekly cisplatin arm than in the 3‐weekly cisplatin arm (84.1% vs. 88.9%).^17^ We, therefore, propose that the weekly administration of cisplatin at 40 mg/m^2^ may have demonstrated non‐inferiority to the 3‐weekly regimen by reducing the impact of nephrotoxicity, allowing the maintenance of a higher dose intensity, and eliciting a more pronounced radiosensitizing effect, even though the cumulative dose was slightly lower. In future studies, not only cumulative cisplatin dose but also cisplatin dose intensity should be considered, particularly when examining dosing schedules.

Despite the findings, this study has some limitations. First, patients who developed AKI may be influenced by additional factors that potentially reduce OS, such as age, pretreatment creatinine, hypoalbuminemia, hypomagnesemia, hypertension, and diabetes mellitus.^13^, ^30^, ^31^, ^32^ Indeed, our separate supplementary analysis revealed that age, pretreatment CCr, and hypoalbuminemia were risk factors for AKI in this setting.^33^ However, as stated, these were supplementary analyses using a randomized phase II/III study to ensure balance between the treatment arms, as demonstrated in Table 1. Second, there is uncertainty regarding the accurate prediction of high‐risk patients who will develop AKI. Supplementary analyses are currently being conducted to address this issue. Third, the reasons for cisplatin dose reduction or discontinuation were not limited to AKI. There were numerous cases in which consecutive dosing for 7 weeks was not feasible owing to reasons other than AKI, such as bone marrow suppression. However, in the weekly arm, no difference was found in the number of cisplatin doses or cumulative doses administered based on the occurrence of AKI; therefore, it was not considered to have a significant impact. Finally, the results pertain to postoperative minimal residual disease, and whether the same theory applies to macroscopic lesions in locally advanced cases remains uncertain. Nonetheless, we demonstrated that the development of AKI influenced the OS of patients diagnosed with HNSCC who underwent postoperative CRT in the 3‐weekly arm but not in the weekly arm. Moreover, we propose that consistent exposure to maintain dose intensity of cisplatin through weekly administration may lead to improvements in survival outcomes.

## 5 CONCLUSIONS

This supplementary analysis of JCOG1008 data revealed that the development of AKI affected the OS of patients diagnosed with HNSCC who underwent postoperative cisplatin‐based CRT in the 3‐weekly arm but not in the weekly arm. Weekly administration can improve kidney safety, ensure the maintenance of cisplatin treatment intensity, and consequently improve long‐term outcomes. Our results provide additional support for the weekly use of cisplatin at a dose of 40 mg/m^2^ in this setting.

## AUTHOR CONTRIBUTIONS

**Yoshinori Imamura:** Conceptualization (lead); formal analysis (supporting); methodology (lead); resources (equal); visualization (supporting); writing – original draft (lead); writing – review and editing (lead). **Naomi Kiyota:** Conceptualization (equal); data curation (lead); funding acquisition (supporting); investigation (lead); methodology (lead); project administration (lead); resources (equal); supervision (lead); writing – original draft (lead); writing – review and editing (lead). **Makoto Tahara:** Conceptualization (supporting); data curation (lead); funding acquisition (lead); investigation (lead); methodology (lead); project administration (lead); resources (equal); supervision (lead); writing – original draft (equal); writing – review and editing (equal). **Takeshi Kodaira:** Resources (equal); writing – original draft (equal); writing – review and editing (equal). **Ryuichi Hayashi:** Project administration (lead); resources (equal); writing – original draft (equal); writing – review and editing (equal). **Hiroshi Nishino:** Resources (equal); writing – original draft (equal); writing – review and editing (equal). **Yukinori Asada:** Resources (equal); writing – original draft (equal); writing – review and editing (equal). **Hiroki Mitani:** Resources (equal); writing – original draft (equal); writing – review and editing (equal). **Shigemichi Iwae:** Resources (equal); writing – original draft (equal); writing – review and editing (equal). **Naoki Nishio:** Resources (equal); writing – original draft (equal); writing – review and editing (equal). **Yusuke Onozawa:** Resources (equal); writing – original draft (equal); writing – review and editing (equal). **Nobuhiro Hanai:** Project administration (supporting); resources (equal); writing – original draft (equal); writing – review and editing (equal). **Akira Ohkoshi:** Resources (equal); writing – original draft (equal); writing – review and editing (equal). **Hiroki Hara:** Resources (equal); writing – original draft (equal); writing – review and editing (equal). **Nobuya Monden:** Resources (equal); writing – original draft (equal); writing – review and editing (equal). **Masato Nagaoka:** Resources (equal); writing – original draft (equal); writing – review and editing (equal). **Shujiro Minami:** Resources (equal); writing – original draft (equal); writing – review and editing (equal). **Ryo Kitabayashi:** Conceptualization (supporting); data curation (lead); formal analysis (lead); methodology (lead); software (lead); validation (lead); visualization (lead); writing – original draft (equal); writing – review and editing (equal). **Keita Sasaki:** Conceptualization (supporting); data curation (lead); formal analysis (lead); methodology (lead); software (lead); validation (lead); visualization (lead); writing – original draft (equal); writing – review and editing (equal). **Akihiro Homma:** Project administration (supporting); resources (equal); writing – original draft (equal); writing – review and editing (equal).

## FUNDING INFORMATION

This study was supported by the National Cancer Center Research and Development Funds (29‐A‐3, 25‐B‐2, 2020‐J‐3, 2023‐J‐03), Grant‐in‐Aid for Clinical Cancer Research (H23‐009 and H26‐052) from the Ministry of Health, Labor and Welfare of Japan, Japan Agency for Medical Research and Development (AMED) (JP16ck0106055, JP19ck0106321 and JP22ck0106596).

## CONFLICT OF INTEREST STATEMENT

Yoshinori Imamura: Speakers' Bureau—Daiichi Sankyo/UCB Japan; MSD. Naomi Kiyota: Honoraria—AstraZeneca; Bayer; Bristol‐Myers Squibb Japan; Eisai; Lilly Japan; Merck Serono; MSD; Ono Pharmaceutical Consulting or Advisory Role—Adlai Nortye; Ono Pharmaceutical; Shift Zero Research Funding—Adlai Nortye (Inst); Bayer (Inst); Boehringer Ingelheim (Inst); Bristol‐Myers Squibb (Inst); Lilly (Inst); Ono Pharmaceutical (Inst); Rakuten Medical (Inst). Makoto Tahara: Honoraria—Bayer; Bristol‐Myers Squibb; Eisai; Lilly; Merck Serono; MSD; Ono Pharmaceutical Consulting or Advisory Role—Astellas Pharma; Bayer; Boehringer Ingelheim; Bristol‐Myers Squibb; Eisai; Genmab; Janssen; Lilly; MSD; Nanobiotix; Nektar; Ono Pharmaceutical; Pfizer; Rakuten Medical Research Funding—AstraZeneca (Inst); Bayer (Inst); Bristol‐Myers Squibb (Inst); GlaxoSmithKline (Inst); Lilly (Inst); Merck Serono (Inst); Merck Sharp & Dohme (Inst); Novartis (Inst); Ono Pharmaceutical (Inst); Pfizer (Inst); Rakuten Medical (Inst). Takeshi Kodaira: Speakers' Bureau—Accuray; AstraZeneca; Bristol‐Myers Squibb; Chugai Pharma; Hitachi; Janssen; Merck Serono; Ono Pharmaceutical; Reason Why Co. Ryuichi Hayashi: Consulting or Advisory Role—Rakuten Medical Japan; Research Funding—Japan Agency for Medical Research and Development. Hiroshi Nishino: No Relationships to Disclose. Yukinori Asada: No Relationships to Disclose. Hiroki Mitani: No Relationships to Disclose. Shigemichi Iwae: Consulting or Advisory Role—Merck (Inst) Speakers' Bureau—Bristol‐Myers Squibb Japan; Merck; MSD; Ono Pharmaceutical Research Funding—Ascent Development Services (Inst); GlaxoSmithKline (Inst); Merck (Inst); MSD (Inst); Ono Pharmaceutical (Inst). Naoki Nishio: No Relationships to Disclose. Yusuke Onozawa: No Relationships to Disclose. Nobuhiro Hanai: Honoraria—Bristol‐Meyers Squibb; Covidien; Eisai; Merck; MSD K.K; Ono Pharmaceutical; Rakuten Medical Research Funding—MSD K.K. Akira Ohkoshi: No Relationships to Disclose. Hiroki Hara: Honoraria—Asahi Kasei; Bayer; Bristol‐Myers Squibb; Chugai Pharma; Daiichi Sankyo/UCB Japan; Lilly; Merck Serono; MSD; Ono Pharmaceutical; Taiho Pharmaceutical; Takeda; Yakult Honsha Consulting or Advisory Role—Boehringer Ingelheim; Bristol‐Myers Squibb Japan; Chugai Pharma; MSD; Ono Pharmaceutical Research Funding—ALX Oncology (Inst); Amgen (Inst); Astellas Pharma (Inst); AstraZeneca (Inst); Bayer (Inst); BeiGene (Inst); Boehringer Ingelheim (Inst); Bristol‐Myers Squibb Japan (Inst); Chugai Pharma (Inst); Daiichi Sankyo (Inst); Dainippon Sumitomo Pharma (Inst); Eisai (Inst); Janssen Oncology (Inst); Merck Serono (Inst); MSD (Inst); Ono Pharmaceutical (Inst); Taiho Pharmaceutical (Inst). Nobuya Monden: No Relationships to Disclose. Masato Nagaoka: No Relationships to Disclose. Shujiro Minami: No Relationships to Disclose. Ryo Kitabayashi: No Relationships to Disclose. Keita Sasaki: No Relationships to Disclose. Akihiro Homma: Speakers' Bureau—Bayer Yakuhin; Bristol‐Myers Squibb; Demant; Eisai; Kyorin; Lilly Japan; Meiji Seika Kaisha; Merck; Mitsubishi Tanabe Pharma; MSD K.K; Ono Pharmaceutical; Rakuten Medical Japan; Sanofi; Taiho Pharmaceutical Research Funding—Eisai; Iwasaki Denshi; Kyorin; Mitsubishi Tanabe Pharma; Ono Pharmaceutical; Otsuka; Taiho Pharmaceutical; Torii Pharmaceutical.

## ETHICS STATEMENT

JCOG1008 was approved by the National Cancer Center Hospital–Certified Review Board (CRB3180008).

## PATIENT CONSENT STATEMENT

All patients provided written informed consent.

## PERMISSION TO REPRODUCE MATERIAL FROM OTHER SOURCES

No written permission is necessary.

## CLINICAL TRIAL REGISTRATION

JCOG1008 is registered with the Japan Registry of Clinical Trials (jRCTs031180135).

## Supporting information


**Figure S1.** Kaplan‐Meier curves in the 3‐weekly arm according to the stage of acute kidney injury. Overall survival (A) and relapse‐free survival (B).

## Data Availability

Anonymized individual participant data that underlie the results reported in this Article will not be shared because patient follow‐up will be continued until December 2023. After publication of the final follow‐up, using data as of December 2023, the data that support the findings of this study are available from the corresponding author after deidentification to investigators whose proposed data use has been approved by investigators of the JCOG Head and Neck Cancer Study Group identified for that purpose. Proposals should be directed to nkiyota@med.kobe-u.ac.jp.
