# Supplementary material for: Effect of acute kidney injury and overall survival in patients with postoperative head and neck cancer who received chemoradiotherapy with cisplatin: A supplementary analysis of the phase II/III trial of JCOG1008
Source: Cancer Med. 2024 Sep 30;13(18):e70235. doi: 10.1002/cam4.70235 (PMC11441389; doi:10.1002/cam4.70235)
Supplement: Supplementary file 1 — Figure S1. Kaplan‐Meier curves in the 3‐weekly arm according to the stage of acute kidney injury. Overall survival (A) and relapse‐free survival (B). [file CAM4-13-e70235-s001.docx]

**Supplementary Materials**


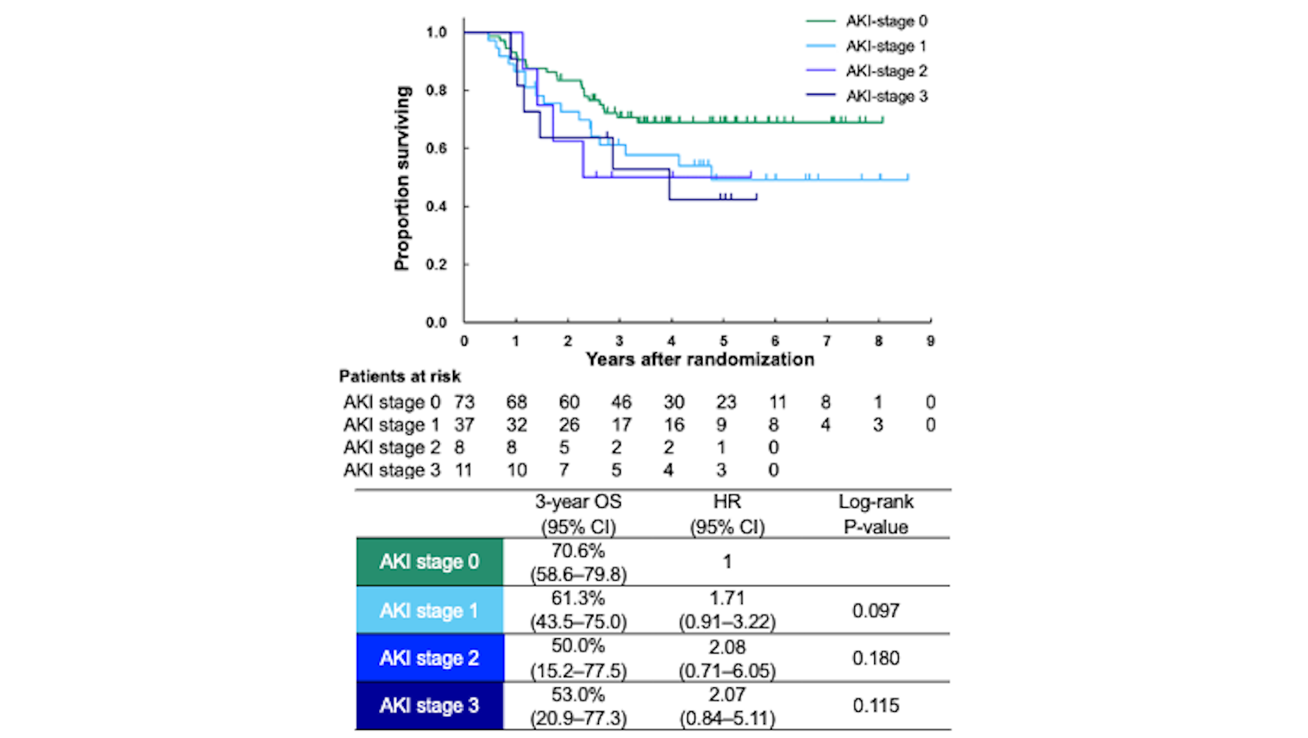

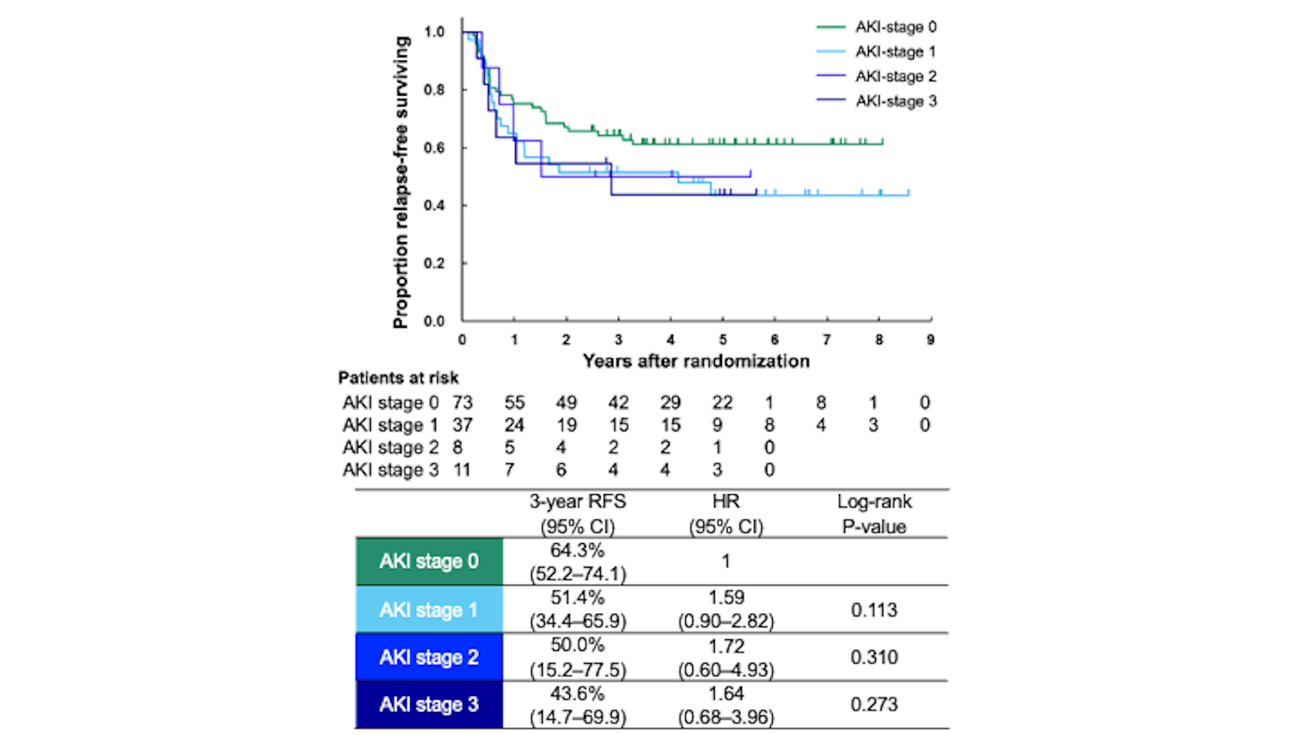


(A) Overall survival, 3-weekly arm (B) Relapse-free survival, 3-weekly arm

**Figure S1**. Figure S1. Kaplan-Meier curves in the 3-weekly arm according to the stage of acute kidney injury. Overall survival (A) and relapse-free survival (B).

**Table S1**. Criteria for reduction or discontinuation of cisplatin

| **Creatinine clearance (mL/min)** | **3-weekly arm** | **Weekly arm** |
| --- | --- | --- |
| ≥ 60 | 100 mg/m^2^ | 40 mg/m^2^ |
| ≥ 50 | 80 mg/m^2^ | 40 mg/m^2^ |
| ≥ 40 | 60 mg/m^2^ | 30 mg/m^2^ |
| ≥ 30 | - | 20 mg/m^2^ |
| < 30 | - | - |
